# Predicting Lyme Disease From Patients' Peripheral Blood Mononuclear Cells Profiled With RNA-Sequencing

**DOI:** 10.3389/fimmu.2021.636289

**Published:** 2021-03-08

**Authors:** Daniel J. B. Clarke, Alison W. Rebman, Allison Bailey, Megan L. Wojciechowicz, Sherry L. Jenkins, John E. Evangelista, Matteo Danieletto, Jinshui Fan, Mark W. Eshoo, Michael R. Mosel, William Robinson, Nitya Ramadoss, Jason Bobe, Mark J. Soloski, John N. Aucott, Avi Ma'ayan

**Affiliations:** ^1^Department of Pharmacological Sciences, Mount Sinai Center for Bioinformatics, Icahn School of Medicine at Mount Sinai, New York, NY, United States; ^2^Lyme Disease Research Center, Division of Rheumatology, Department of Medicine, Johns Hopkins University School of Medicine, Baltimore, MD, United States; ^3^Department of Genetics and Genomic Sciences, Icahn School of Medicine at Mount Sinai, New York, NY, United States; ^4^Ibis Biosciences (an Abbott Laboratories company), Carlsbad, CA, United States; ^5^Division of Immunology and Rheumatology, Department of Medicine, Stanford University School of Medicine, Stanford, CA, United States

**Keywords:** Lyme disease, PTLDS, PBMCs, machine learning, data mining, RNA-seq

## Abstract

Although widely prevalent, Lyme disease is still under-diagnosed and misunderstood. Here we followed 73 acute Lyme disease patients and uninfected controls over a period of a year. At each visit, RNA-sequencing was applied to profile patients' peripheral blood mononuclear cells in addition to extensive clinical phenotyping. Based on the projection of the RNA-seq data into lower dimensions, we observe that the cases are separated from controls, and almost all cases never return to cluster with the controls over time. Enrichment analysis of the differentially expressed genes between clusters identifies up-regulation of immune response genes. This observation is also supported by deconvolution analysis to identify the changes in cell type composition due to Lyme disease infection. Importantly, we developed several machine learning classifiers that attempt to perform various Lyme disease classifications. We show that Lyme patients can be distinguished from the controls as well as from COVID-19 patients, but classification was not successful in distinguishing those patients with early Lyme disease cases that would advance to develop post-treatment persistent symptoms.

## Introduction

Lyme disease (LD) is a tick-borne illness that has become a growing concern in the United States (US) and Canada. LD spreads exclusively by two tick species in the Northern hemisphere, *Ixodes scapularis* and *Ixodes pacificus*. The disease is caused by the spirochete bacteria *Borrelia burgdorferi sensu stricto* and is transmitted to humans through one of the vector's blood meals (1). Comprising 62.6% of all vector-borne diseases, and 81.19% of all tick-borne disease, *Borrelia burgdorferi sensu stricto* was the most common vector-borne pathogen in the United States from 2004 to 2016 (2). Approximately 30,000 diagnosed cases of LD are reported to the CDC each year, with an estimated true burden of ~300,000 cases (3, 4) and yearly healthcare cost of ~$1 billion in the US (5). Cases in the US are concentrated within the Northeast, Mid-Atlantic, Midwest, and coastal West regions (1). Due to the tick's seasonal lifecycle, transmission of the pathogen and subsequent human infection occurs at higher rates in spring, summer, and the early part of autumn when the various life-stages of the vector quest for a meal (6). Once infected, initial onset of symptoms can manifest ~7–14 days after transmission, although both earlier and later initial onset has been documented (6).

Clinical demonstration of untreated Lyme disease is divided into three stages: the early localized stage, early disseminated stage, and late disseminated stage (6, 7). The early localized stage is characterized by the erythema migrans (EM), a skin lesion that is often red and round or oval shaped that manifests at the site of the bite, and which can be accompanied by mild flu-like symptoms of fever and fatigue. However, the rash is absent or undetected in ~5–30% of cases (7). Symptoms can progress into the early disseminated stage when the patient lacks initial treatment during the early localized stage and the bacteria disseminate hematogenously to other areas of the skin or other organ systems. Multiple EMs may be present around the body, although they are typically less inflamed than the primary EM at the site of the initial tick bite. In addition, the patient may exhibit Lyme carditis or Lyme neuroborreliosis (1, 7). Approximately 6 months after initial disease onset and when left without treatment, the patient may experience signs of the late disseminated stage, specifically late Lyme arthritis (which may occur in up to 60% of patients with a history of primary EM) or neurologic disease (7). Following appropriate antibiotic treatment, a subset of patients experience a range of persistent, significant, but often non-specific symptoms which frequently include fatigue, widespread musculoskeletal pain, and cognitive difficulties, among others (8). Approximately 10–20% of patients will meet criteria for post-treatment Lyme disease (PTLD), which includes the presence of specific symptoms as well as significant impact of these symptoms on life functioning (9). Testing and diagnosis of LD has proven to be difficult or unreliable. Bacteria cultivation requires specialized medium and frequently results in low-yield cultivation (1). The universally-accepted diagnostic test for LD is a two-tier serological test: a positive enzyme-linked immunosorbent assay (ELISA) test followed by a positive Western blot test for IgM and IgG *B. burgdorferi* antigens (10). Unfortunately, sensitivity is low at 29% during the early localized stage, and the two-tiered serological test is not recommended for early diagnosis in the first few weeks of infection (11). In the absence of a laboratory diagnostic tool, the diagnosis of early LD is reliant on a demonstrated EM that occasionally does not present or is not observed, which can lead many patients to progress to the early disseminated stage and its debilitating symptoms before the disease is diagnosed and treated.

To further our understanding of the molecular mechanisms that lead to LD symptoms, we examined the longitudinal changes in gene expression as a tool for deep phenotyping of diagnosed LD patients and healthy controls. RNA-sequencing profiling was administered from peripheral blood mononuclear cells (PBMCs) collected during multiple patient visits over a 1-year timespan. Deep phenotyping was performed by examining the projection of RNA-seq data into lower dimensions for unsupervised clustering of the patients based on their gene expression vectors. In addition, differential gene expression analysis followed by enrichment analysis was employed to identify upstream regulatory mechanisms and disease phenotypes associated with LD. This analysis was augmented with single cell deconvolution analysis. Since we observed clear separation between controls and cases, we developed several classifiers as potential diagnostic tools and for the further identification of molecular mechanisms underlying LD phenotypes. These include predictors that distinguish healthy controls from Lyme patients, Lyme patients from COVID-19 patients, and whether Lyme patients will advance to develop persistent symptoms.

## Materials and Methods

### Patient Recruitment

The current study is part of a larger, ongoing prospective cohort study of patients with LD and non-LD controls. Adult patients with early LD were predominantly recruited from primary or urgent care settings in the Mid-Atlantic area of the United States. All participants were required to have a physician-documented EM of >5 cm present at the time of enrollment, thereby meeting CDC criteria for “confirmed” LD (12). Although early LD patients were eligible with up to 72 h of appropriate antibiotic exposure at the time of enrollment, the majority (63.0%) were antibiotic-naive at their first visit. Patients with a prior history of LD or those who had received the LD vaccine were excluded from the study. Patients were also excluded for a range of self-reported prior medical conditions associated with significant immunologic impact and/or subjective symptoms which may overlap with PTLD: chronic fatigue syndrome, fibromyalgia, unexplained chronic pain, sleep apnea or narcolepsy, autoimmune disease, chronic neurologic disease, liver disease, hepatitis, HIV, cancer or malignancy, major psychiatric illness, or drug or alcohol abuse. Non-Lyme infected controls were recruited from similar care settings as LD cases, as well as via community recruitment using flyers and online advertising. Controls underwent an initial screening two-tier antibody test and were required to test negative prior to enrollment. Follow-up tests were conducted at each subsequent time point, with all controls testing two-tier negative for the duration of the study. Controls were also screened for the same prior medical history conditions as cases and were also required to be free of any history of prior clinical LD. Participants with LD and non-LD controls were followed over multiple visits up to 1 year after study entry (Table 1). The Institutional Review Board of the Johns Hopkins University School of Medicine approved this study, and all participants signed written consent prior to initiation of any study activities.

**Table 1:** Breakdown of patient counts by visit.

**Visit**	**Cases**	**Controls**
Visit 1	Baseline (diagnosis) *n* = 72	Baseline *n* = 44
Visit 2	3 weeks (end of treatment) *n* = 73	N/A
Visit 3	6 months *n* = 62	6 months *n* = 39
Visit 4	1 year *n* = 61	1 year *n* = 25
Visit 5	N/A	2 years *n* = 24

### Clinical Data Collection

A trained interviewer administered a series of detailed questionnaires regarding demographics and general medical, medication, and symptom histories to both patients with early LD and controls at each study time point. In addition, specific data were gathered from patients with early LD regarding their acute illness. At the follow-up study visits, participants self-administered a 36-item symptom list developed based on prior clinical and research experience among patients with Lyme disease. The presence or absence of self-reported fatigue, musculoskeletal pain, and/or cognitive difficulty at V3 and V4 were used to classify participants into those with persistent symptoms and those without. Given the relatively small number of participants in this analysis, we did not require participants to also meet criteria for functional impact and therefore those with persistent symptoms did not meet the full PTLD case definition. During the first study visit, a sensitive, PCR-based approach (PCR/ESI-MS), as previously described in a subset of these participants (13), was used to detect the presence of *B. burgdorferi* in the skin (Supplementary Figure 1A) and blood (Supplementary Figure 1B).

### PBMC Isolation and Library Generation

PBMCs were isolated from fresh whole blood using Ficoll (Ficoll-Paque Plus, GE Healthcare) and total RNA was extracted from 10^7^ PBMCs using RLT Lysis Buffer (Qiagen) by following manufacturer's instructions. The NEBNext Ultra II Directional RNA Library Prep Kit for Illumina (Cat# E7765) was used to generate RNA-seq libraries. Briefly, Poly A RNAs were isolated from total RNAs using NEBNext Poly(A) Magnetic Isolation Module (NEB #E7490) and then fragmented for cDNA synthesis. End repair is performed where 3' to 5' exonuclease activity of enzymes removes 3' overhangs and the polymerase activity fills in the 5' overhangs. An “A” base is then added to the 3' end of the blunt phosphorylated DNA fragments which prepares the DNA fragments for ligation to the sequencing adapters, which have a single “T” base overhang at their 3' end. Ligated fragments are subsequently size-selected through purification using the Sample Purification Beads included in the kit and undergo PCR amplification to prepare the “libraries.” The BioAnalyzer is used for quality control of the libraries to ensure adequate concentration and appropriate fragment size free of adapter dimers. The resulting library insert size is 200–500 bp with a median size around 300 bp. Libraries were uniquely barcoded and pooled for HiSeq2500 sequencing.

### RNA-Seq Data Processing

Raw RNA-seq FASTQ files were processed by FastQC, a quality control tool for high throughput sequencing data (14). The samples were aligned to the human genome (hg38) with the STAR RNA-seq aligner (15). Picard tools were then used for manipulating the output from STAR so it can be piped into featureCounts (16) for gene, exon, and transcript quantification. This pipeline was encoded in python and it is made available on GitHub at https://github.com/lymeMIND.

### Cytokine/Chemokine Assays

The levels of 38 cytokines and other immune mediators were measured using Bio-Plex cytokine arrays and the Bio-Plex 200 System (Bio-Rad Laboratories). All tests at the participant level for each specific immune mediator were run by the same system. All tests were run as recommended by the manufacturer using previously described optimized assay protocols (17). The cytokines, chemokines, and acute phase markers measured were: Eotaxin, FGF basic, G-CSF, GM-CSF, HTARC, IFN-γ, IL-1β, IL-1rα, IL-2, IL-4, IL-5, IL-6, IL-7, IL-8, IL-9, IL-10, IL-12, IL-13, IL-15, IL-17, IL-17A, IL-17F, IL-21, IL-22, IL-23, IL-25, IL-31, IL-33, IP-10, MCP-1(MCAF), MIP-1α, MIP-1β, MIP-3β, PDGF-ββ, RANTES, sCD40L, TNF-α, and VEGF. Data processing was performed using Bio-Plex manager software version 4.4.1, and serum concentrations were interpolated from standard curves for each respective cytokine. These data were transformed using the log of the “ratio to average” for ease of interpretation. This was calculated by setting all values <1–1 pg/mL, and then calculating the log base 2 of [(value)/(average value in the cohort)]. As a result, 0 represents an average value, 1 represents a value which is 2 times the average, and 2 represents a value which is 4 times the average.

### Data Analysis and Visualization

The RNA-seq gene counts were log2 transformed, z-scored, and quantile normalized (18) across all patients irrespective of time-point. These features were used for the subsequent data visualizations and classifiers. For the differential expression analyses, RNA-seq gene counts were quantile normalized across all patients. Uniform Manifold Approximation and Projection (UMAP) (19) was used to perform non-linear manifold aware dimensionality reduction and is used for visualizing the features for all samples in two dimensions. Samples were clustered using the k-means clustering algorithm on the UMAP manifold. Silhouette clustering analysis (20) was applied to identify the optimal number of clusters. Comparisons by cluster were performed using Kruskall-Wallis tests for overall *p*-value, and Wilcoxon rank sum test for individual pairwise comparisons for continuous variables, and chi-square test for categorical variables.

The log2 transformed RNA-seq gene count features were selected in several ways and used to train and test a series of Random Forest (21) and Logistic Regression classifiers. We employed the same approach to distinguish cases from controls, as well as Lyme patients with persistent symptoms from those without at later time points Samples were randomly grouped into a stratified training and test set using 75 and 25% of the samples, respectively, while preserving the ratios of positive and negative labels in each group. The Random Forest classifier was trained using all features to distinguish patients. First, the training was done in such a way that would lead to a fully grown and unpruned set of decision trees, and then in a way that constrains the tree to utilize the most informative top 50 features across all trees. The Logistic Regression classifier was trained using all the features to distinguish patients. The top 50 features with the highest absolute slopes after training were then used to train another Logistic Regression classifier with access to only those features. Another Logistic Regression classifier was trained using the top 50 features with the highest ANOVA F-test. We computed the unit mean and standard deviations from the log2 normalized training data and applied z-score normalization to the log2 normalized training and test data. Performance of each of the models on the test sets were collected and visualized using AUROC and PR curves.

CIBERSORT was used along with the benchmarked LM22 signature file, which contains signatures for 22 immune cell types to derive cell type ratios from our patient's RNA-seq gene counts (22). The resulting cell type ratios were investigated for statistically significant feature correlations and the features themselves were used with the Random Forest classifiers and the Logistic Regression classifiers to distinguish Lyme patients from healthy controls, and Lyme patients with persistent symptoms from those without. Our RNA-seq counts were further contrasted with the RNA-seq counts from a study of the immune responses in several RNA-seq analyzed PBMCs of patients with COVID-19 (GSE152418). The data were merged by independently log2-transformed, z-score normalized, and quantile normalized before being combined into a unified set of gene expression and corrected for batch effects which distinguished the datasets using ComBat (23).

Analyses and figures were produced using python scikit-learn and the scipy ecosystem, SAS Software (version 9.4; SAS Institute Inc., Cary, NC, USA), and GraphPad Prism (version 8.1.0; GraphPad Software, San Diego, CA, USA).

## Results

### RNA-Seq Processing, Clustering, and Visualization

**Figure 1:**
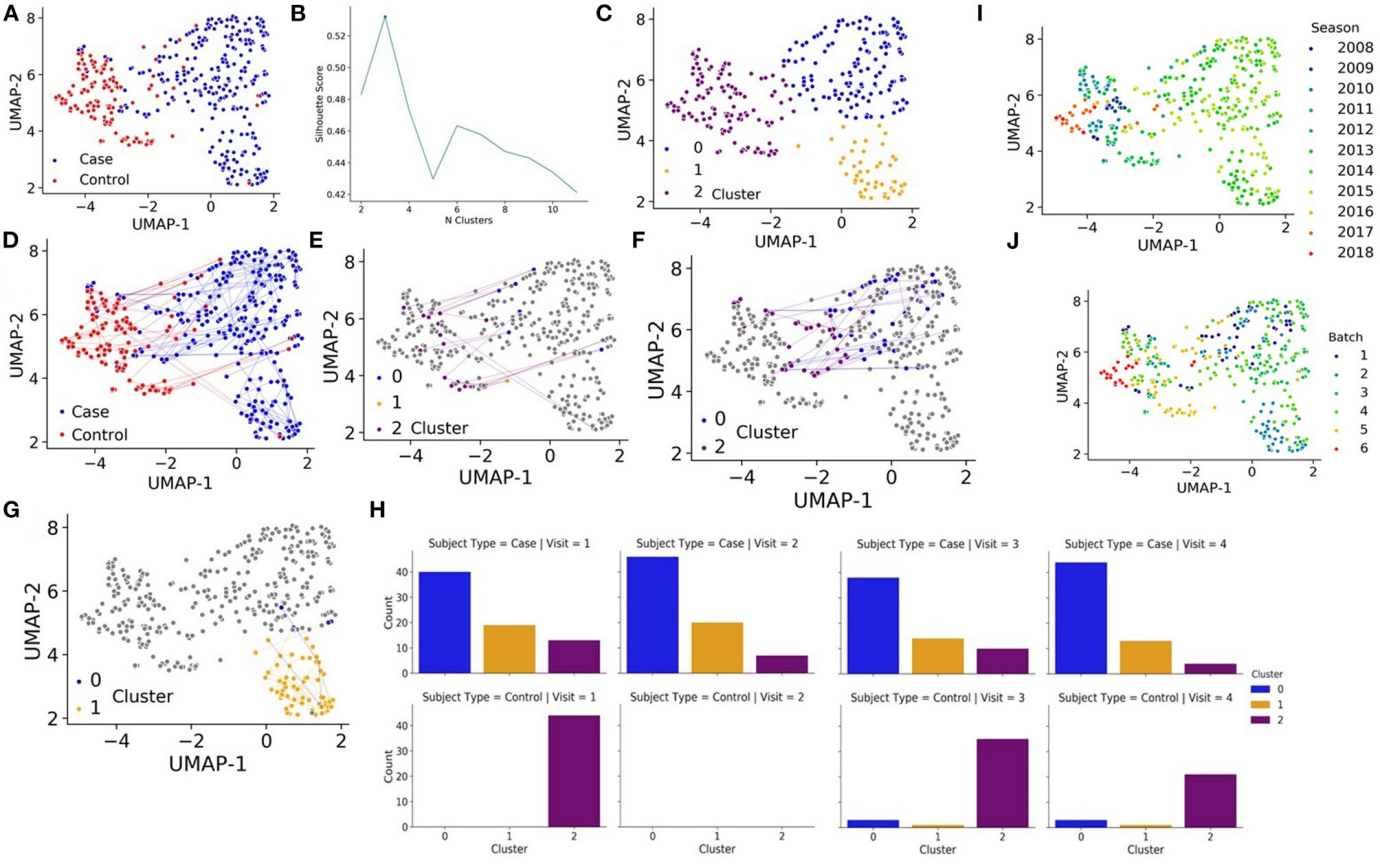
**(A)** UMAP projections of all samples collected for the study based on RNA-seq expression where each sample, representing a patient at a specific visit, is colored by cases (blue) vs. controls (red). **(B)** K-means is fit to the quantile normalized gene counts matrix and silhouette analysis is performed to identify an optimal number of clusters. **(C)** UMAP projections of all samples collected for the study based on RNA-seq expression where each sample, representing a patient at a specific visit, is colored by automatic cluster assignment. **(D)** UMAP projections of all samples collected for the study based on RNA-seq expression where each sample, representing a patient at a specific visit, is colored by cases (blue) vs. controls (red) and line trace the trajectory of patients over time. **(E)** UMAP projections of controls that change clusters (in color), cluster membership (color) and line trace of patients over time. **(F)** UMAP projections of cases that change clusters from cluster 0 (in color), cluster membership (color) and line trace of patients over time. **(G)** UMAP projections of cases that change clusters from cluster 1 (in color), cluster membership (color) and line trace of patients over time. **(H)** Membership of cases (left) and controls (right) for each visit in each automatically detected cluster. **(I)** UMAP projections of all samples colored by the season/year when the data was collected. **(J)** UMAP projections of all samples colored by one of six batches.

Seventy-two cases (41 males and 31 females) diagnosed with early localized and early disseminated LD along with 44 controls (19 males and 25 females) were enrolled in the longitudinal study. After aligning the RNA-seq data, transcript counts were converted to the gene level and then counts were log transformed and normalized (see methods). We then applied Uniform Manifold Approximation and Projection (UMAP) (19) to estimate similarities and differences between the samples, each representing a patient at a specific visit. Interestingly, cases were mostly separated from the controls, even after 6 months and 1 year follow up visits (Figure 1A). Such separation cannot be attributed to batch, gender, or seasonal effects (Supplementary Figure 2). Next, we aimed to automatically identify clusters based on the samples' RNA-seq data to see if the cases and controls further cluster into subtypes. The Silhouette score identified an optimal number of 3 clusters (Figure 1B). The automatic clustering placed the controls in cluster 2. LD cases are divided into two distinct clusters, clusters 0 and 1, with a smaller group of cases clustering with the controls in cluster 2 (Figure 1C). There are two notable observations. First, most cases and controls remain in one cluster over the course of study (Figures 1D–G). Secondly, some cases that are mainly in cluster 0 move around to cluster 2 (mostly controls) (Figure 1F), but there are no patients from cluster 1 that return to the control cluster 2 even after 1 year (Figures 1G–H). This is surprising because most LD patients that receive timely treatment are expected to return to a normal state after several weeks. We observe that none of the clinical measured variables can clearly explain the overall long-lasting immune activation in the LD cases. However, it should be noted that the data collected for this study was processed in batches over several years (Figures 1I–J). If we plot the data by batch or by year, we see clear patterns that separate the controls from most of the cases (Supplementary Figure 2). While it is possible that there are some batch effects within the data, such separation cannot explain the clear long-term immune activation observed for the cases. We also attempted to remove these batch effects with the ComBat method (23) and the results regarding long-term immune activation remain.

### Lyme Serology Projection and Cytokine Profiling

**Figure 2:**
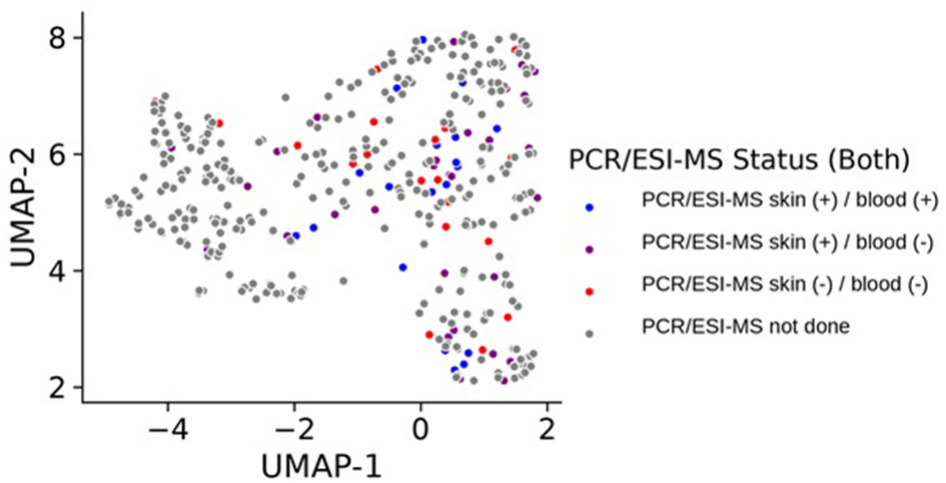
UMAP projection of all samples collected for the study based on RNA-seq expression where each sample, representing a patient at a specific visit, is colored by detection status of Borrelia antigens in their blood or skin. Samples colored in gray did not have data on PCR/ESI-MS status either because they represent control samples, or because they represent case follow-up time points where these data were not obtained.

**Figure 3:**
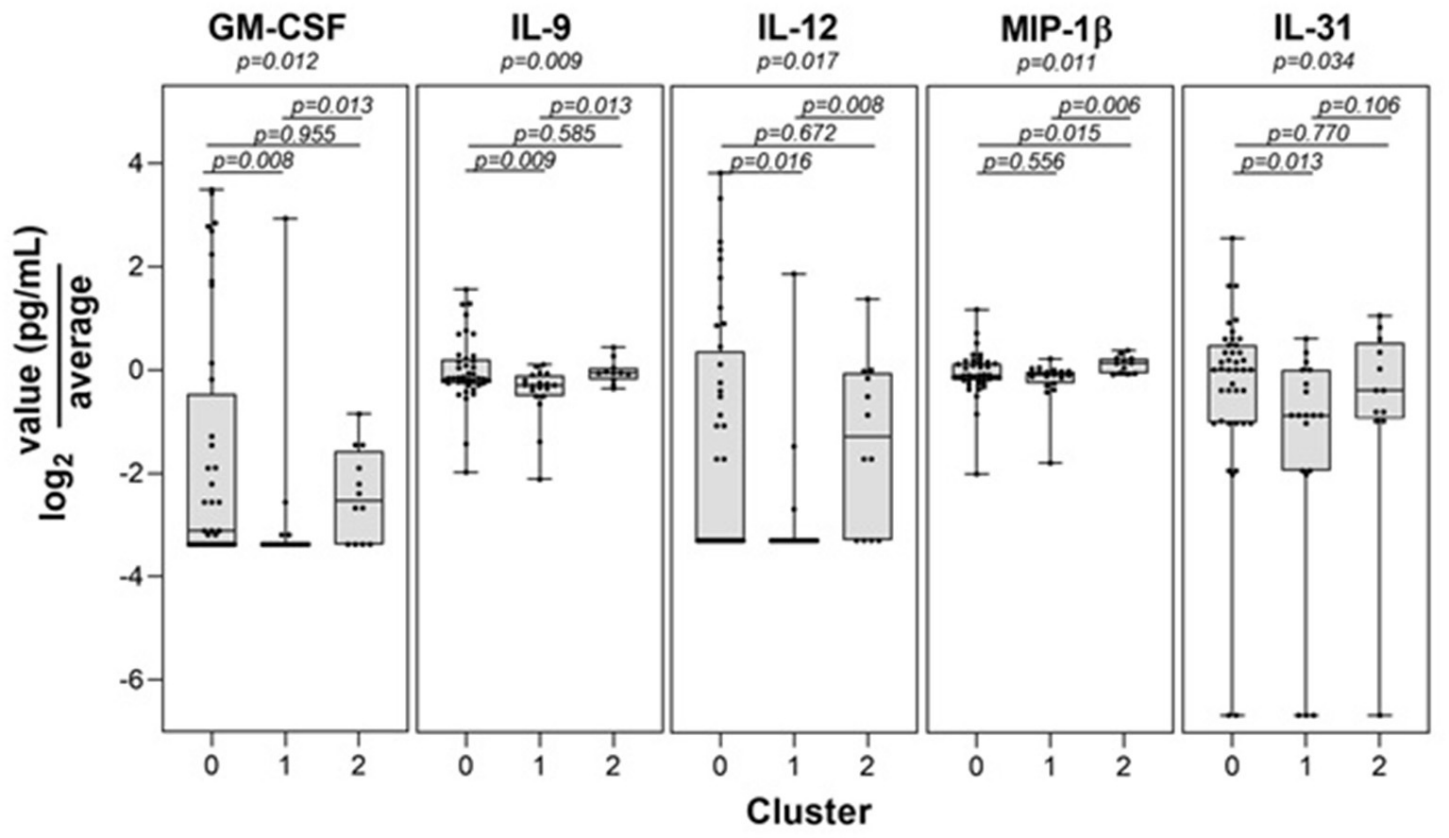
Statistically significant immune mediator differences among transcriptome-defined clusters. Levels of the 5 immune mediators found to be statistically significant by cluster are shown.

Control participants generally remained in cluster 2 over time, with only 9 of the 132 (6.8%) total control visits found in clusters 0 or 1. We compared those control study visits in cluster 2 with those in clusters 0 or 1 and did not find statistically significant differences in Lyme serology (ELISA values, or the number of reactive IgM or IgG bands), or the percent reporting a recent viral infection such as a cold or flu in the past 10 days (*p* > 0.12 for each comparison). In addition, there were no new diagnoses or tick bites in the preceding interval reported by controls at any of the non-cluster 2 study visits. Direct evidence of infection was obtained using a sensitive PCR/ESI-MS approach that detected *Borrelia burgdorferi* in the skin or blood of most of our patients. Interestingly, there was a subgroup of cases that were PCR/ESI-MS negative and who failed to seroconvert on standard two-tier antibody tests [Figure 2, as previously identified in (13)]. Remarkably, these patients generally cluster within the other cases, suggesting that these patients may have LD, but current assays are not capable of detecting the presence of Borrelia in their skin or blood. It should be noted that two patients were clinically diagnosed with LD and considered cases but have negative detection of Borrelia antigens and clustered with the controls. These patients may represent a Lyme look-alike group. Thirty-eight cytokines and chemokines were measured in the sera of cases and controls and 5 were statistically significant by cluster group (*p* < 0.05, see Figure 3), while 7 were borderline statistically significant (0.10 < *p* < 0.05: IL-23, IL-7, IL-13, IL-15, PDGF-ββ, IL-21, and IL-17F). The remaining *n* = 26 was not statistically significant by cluster group. Individual pairwise comparisons were also conducted between clusters.

### Differential Gene Expression Analysis

**Figure 4:**
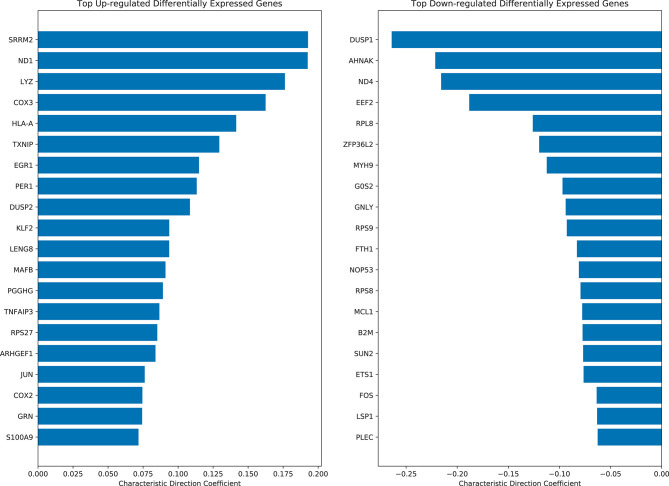
Top ranked up-regulated and down-regulated differentially expressed genes in LD cases based on the characteristic direction method.

Differential expression analysis was performed using the Characteristic Direction (CD) method (24) on all quantile normalized case and control samples. Interestingly, three of the top-ranked up-regulated genes in LD cases are the human leukocyte antigens HLA-A and HLA-B, which suggest inflammation via adaptive immune activation (Figure 4). The top 200 up-regulated and down-regulated genes were subjected to enrichment analysis with Enrichr (25) to identify biological processes and pathways that are altered in the LD cases. As expected, enrichment terms associated with the immune response are associated with the top 200 up-regulated genes in LD cases (Supplementary Figure 3). Specifically, NFkB/RelA is consistently detected as the most enriched transcription factor based on ENCODE (26), TRRUST (27), TRANSFAC (28) and JASPAR (29), and Transcription Factor protein-protein interactions (PPIs). The most enriched pathways are those associated with Influenza, Salmonella, and Ebola infections. Interestingly, arthritis is the top enriched term using the Jensen DISEASES (30) library. Arthritis is a known symptom for LD patients (31). LINCS (32) L1000 (33) ligand perturbations up-regulated genes are enriched for signatures for IL-1 and TNF alpha, supporting immune system activation; and dbGAP (34) enrichment analysis points to leprosy and gout as the top terms. The mycobacterium tuberculosis is the most enriched term for Microbe Perturbations from GEO up library, suggesting that Borrelia effects on gene expression might be most like the effects of this pathogen.

The most striking enrichment result is the top enriched term returned from the “Rare Diseases AutoRIF ARCHS4 Prediction” gene set library which is Erythema elevatum diutinum. This library contains 3,725 gene sets created using the Geneshot (35) tool to search the names of various rare diseases on PubMed. Geneshot uses AutoRIF, which is a resource containing PubMed IDs and the genes mentioned in the title and abstract of these publications. For each rare disease term, the associated PubMed IDs are cross referenced to the AutoRIF resource and ranked by publication frequency. Geneshot then converts these generated gene lists into predicted gene lists using co-expression data from ARCHS4 (36) to identify genes that may be associated with disease terms but not yet studied or published in the literature. All 44 genes associated with Erythema elevatum diutinum were contained within the top 200 positive top ranked genes from the differential expression analysis. These results suggest that erythema disease genes are associated with an immune module that is highly relevant to the molecular mechanisms of LD.

We also examined the differentially expressed genes (DEGs) that were driving the separation of the LD cases into two clusters. Not surprisingly, each of these two clusters of cases displayed unique and defining sets of DEGs (Supplementary Figure 4). The top 200 up-regulated and down-regulated genes in each cluster was subjected to enrichment analysis to identify biological processes and pathways that are unique to each cluster. Surprisingly, HLA-E, a class Ib molecule that can serve as regulatory ligand for NK cells was prominently down-regulated in cluster 0 when compared to cluster 1. These and other enriched terms, derived from the up-regulated genes in clusters 0 vs. 1, and account for the majority of Lyme cases, are visualized as bar charts (Supplementary Figure 5). These two clusters share features that include T cell receptor signaling, the involvement of monocytes and CD4+ T cells, enrichment of Rel and NFKB transcription factors and association with arthritis and leprosy. Cluster 0 is distinct in that monocytes, dendritic cells, and CD8+ T cell involvement are implied. Distinct features of cluster 1 include neutrophils, IL-4 signaling and IgE/IgA synthesis. As above, the most remarkable enrichment came with results from the “Rare Diseases AutoRIF ARCHS4 Prediction” gene set library with each cluster associated with distinct diseases. The rare diseases associated with cluster 0 include those with autoimmune or inflammatory features while cluster 1 rare diseases are largely driven by non-immune mechanisms. This highlights the differences between patients within these two clusters. Such differences can potentially be explained by different types of cytokine CD4+ helper T cells (Th) response.

### Cell Type Deconvolution With CIBERSORT

**Figure 5:**
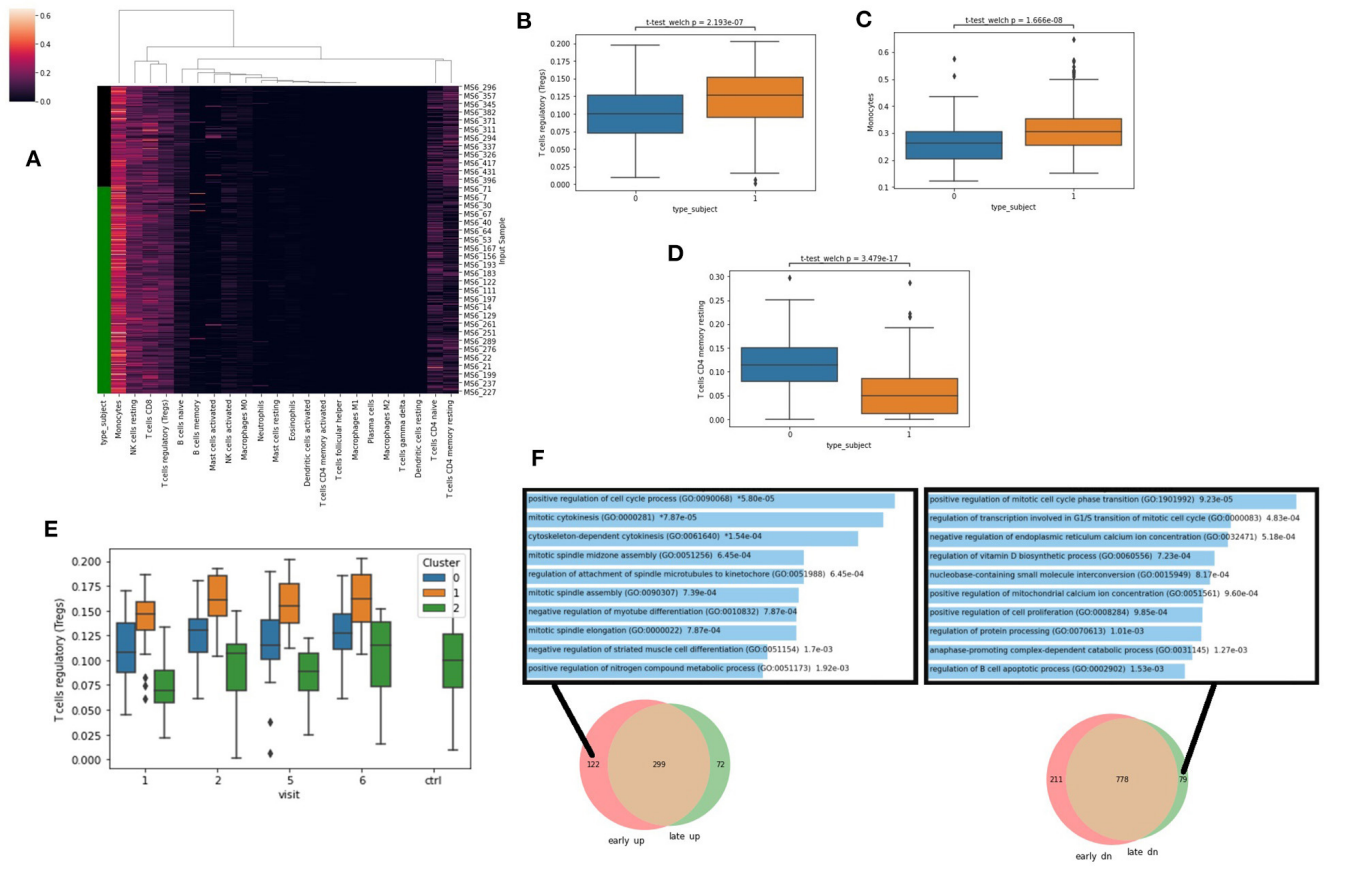
**(A)** Cell-type ratios were derived from gene expression with CIBERSORT. Green bar denotes Lyme disease patients. **(B–D)** Cell-types ratios with significant differences across all visits in healthy patients (type_subject = 0) vs. those with Lyme disease (type_subject = 1) Significance computed with Welch's unequal variance *t*-test for independence. **(E)** Predicted levels of Tregs at each visit for each cluster based on CIBERSORT LM22. **(F)** Comparing the identified DEGs between cases and control and early and late time-points. Enrichment analysis with Enrichr was applied to the unique genes that are only up early (left), or only down (dn) late (right) with the gene ontology biological processes gene set library.

To further explore and extract information from the RNA-seq data, we next applied deconvolution algorithms to identify potential changes in cell type composition between the controls and cases. Specifically, we applied the CIBERSORT algorithm with the LM22 reference signatures (22) which identifies the proportion of 22 immune cell types in a sample (Figure 5A). We observe that the Lyme disease cases have significantly more Tregs (Figure 5B), more monocytes (Figure 5C), and less resting T memory cells (Figure 5D). These observations suggest a general activation of an immune response consistent with the conclusion made via the differential expression analysis. Interestingly, the increase in Tregs compared with the controls is more pronounced in cluster 1 compared with cluster 0 across all visits (Figure 5E). This further supports the overall potentially more robust immune response for the patients within cluster 1. Tregs are known to increase with infection and inflammation. They suppress auto-immune host tissue damage and assist establishing tolerance when the inflammation resolves. Next, we asked whether there are gene modules that return to normal level over time and approach the control subjects, and conversely others that do not. For this, we created two Venn diagrams that highlight the unique genes that are up/down early and those that are up/down late when comparing the cases to the controls. We observe that many cell cycle genes are uniquely up regulated at early visits and down regulated at late visits (Figure 5F). This is consistent with the known rapid proliferation of T cells that occurs at the initiation of innate immune response, and apoptosis and other regulatory mechanisms during adaptive immunity stages.

### Machine Learning Applications to Classify Patients

**Figure 6:**
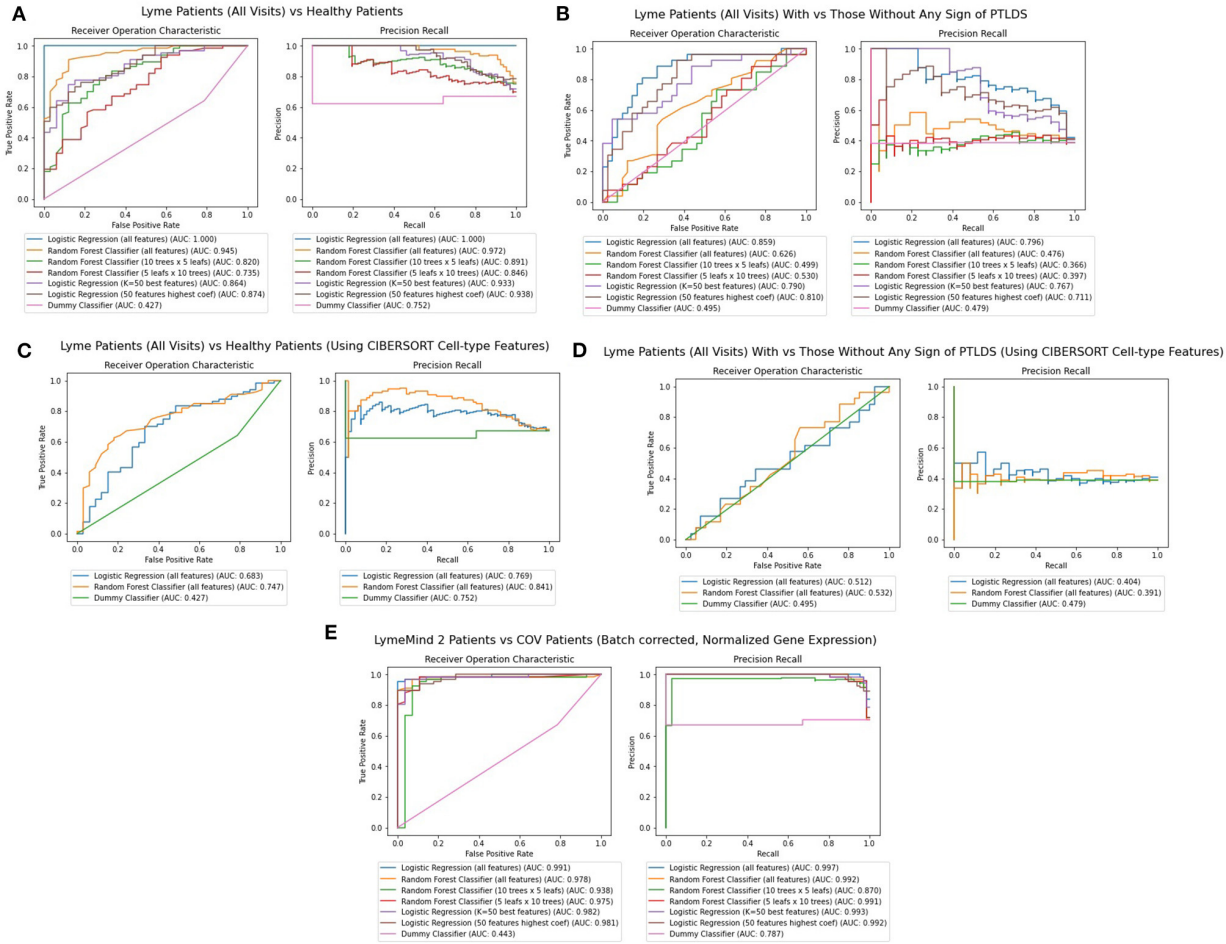
**(A)** AUROC and PR curves for classifiers predicting whether a gene expression sample is from a Lyme disease diagnosed patient or a healthy control across all visits. **(B)** AUROC And PR curves for classifiers predicting whether a gene expression sample is from a Lyme disease diagnosed patient with persistent symptoms. **(C)** AUROC and PR curves for classifiers predicting whether a gene expression sample is from a Lyme disease diagnosed patient or a healthy control across all visits using CIBERSORT features instead of RNA-seq gene expression. **(D)** AUROC and PR curves for classifiers predicting whether a gene expression sample is from a Lyme disease diagnosed patient with persistent symptoms using CIBERSORT features instead of RNA-seq gene expression. **(E)** AUROC and PR curves for classifiers predicting whether a patient's observed gene expression, normalized and batch effect corrected across experiments, suggests they have Lyme disease or COVID-19.

Finally, we applied machine learning methods to attempt to diagnose patients based on their RNA-seq gene expression profiles. Random Forest and Logistic Regression classifiers were trained using stratified training and testing data accounting for 75 and 25% of the RNA-seq from Lyme disease and control samples, respectively. These features are RNA-seq gene expression counts that were log2-normalized, variance-filtered, z-score and quantile normalized. Overall, five different classifiers were constructed: (1) a classifier for predicting whether a patient has Lyme disease, or is an healthy control (Figure 6A), (2) a classifier for predicting whether a patient that has Lyme disease will progress to having persistent symptoms (Figure 6B), (3) and (4) classifiers that attempt to perform the same classifications as 1 and 2, but with CIBERSORT transformed features instead of using the gene expression features directly (Figures 6C,D); and finally, (5) a classifier for predicting whether a patient has Lyme disease or COVID-19 (Figure 6E). The 17 COVID-19 RNA-seq samples were taken from a recent study that profiled patients' PBMCs (GSE152418). We evaluate the performance of each of the trained classifiers instance by computing AUROC and Precision-Recall curves (Figure 6). After five passes of this procedure, we aggregate these metrics to provide a sense of performance and generalizability of the classifiers tested. Overall, we observe that the Logistic Regression classifiers outperform the Random Forest classifiers when using all genes or when restricting the classifiers to use the top 50 most informative genes. The performance of these classifiers suggests that they could potentially aid in diagnosis but may not be good enough to replace existing methods. Additional information about the population of Lyme disease patients would be necessary to adequately assess the performance of this approach in a clinical setting. In addition, more samples and uniform processing of the data will be required. We should also note that the comparison to the COVID-19 patients has several caveats. For one, we are comparing acute viral infection to persistent bacterial infection. The data from the COVID-19 study and our LD study were applied to different populations. These studies were conducted by different groups that employed different protocols to collect and process the data.

### Web supplement

To make the data, analysis, and results from this study accessible and reusable, we developed two web supplement components, one is a Jupyter Notebook with the code, markdown text, and figures generated for this study. The other is an interactive plot that provides enrichment analysis for the different clusters. These two web-based components can be accessed from: https://commons.lymemind.org/#/Notebook and https://commons.lymemind.org/#/Viewer.

## Discussion

The molecular mechanisms underlying the disease course and outcomes of LD are still poorly understood. In this study, we applied in-depth examination of RNA-seq profiling of 73 LD patients over a period of 1 year and compared these to 44 non-LD infected controls over a period of 2 years. The gene expression analysis clustered controls and LD cases into three distinct clusters, each with distinct clinical and immunological features. The majority of cases remained within a single cluster even up to 1 year after diagnosis. Enrichment analysis of the differentially expressed genes between the cases and controls identified up-regulation of immune response genes as well as genes specific for diseases displaying erythema. The clear separation between controls and cases enabled us to develop expression-based machine learning classifiers that may inform improved diagnosis. In fact, we developed three different types of classifiers. One to discriminate between LD cases and healthy controls, one to distinguish between LD cases and those cases that will progress to having persistent symptoms, and one that to classify LD from COVID-19 patients. Overall, the results from these classifiers are encouraging. The cost of RNA-seq is continually dropping so it could be used as a practical approach. While it is difficult to tell whether such a classifier will be able to discriminate from other similar bacterial infections, the up-regulation of immune response genes observed in the cases can be readily detected via expression profiling of their PBMCs.

Two other prior studies attempted to use genome-wide gene expression data to profile LD patients (37, 38). Both studies profiled gene expression from PBMCs extracted from LD patients and controls. Both studies had less patients compared with the number of patients profiled in our study. The size of the cohorts is critical to gain statistical insights and identify patterns in the data collected from these patients. The first study (37), analyzed acute LD patients using RNA-seq profiling and the conclusions of this study are similar to what we observed, which is an increase in immune response genes in the cases and continued dysregulation of gene expression months post infection and treatment. However, it was unknown whether patients return to normal after several months from their initial diagnosis.

The second more recent study (38), was a longitudinal study using cDNA microarrays to profile gene expression and, unlike ours and other findings (37), found that the DRGs identified during the acute phase returned to normal levels at 6 months. There are several potential reasons for this difference. First, this study was conducted using a smaller number of LD cases, with 10 analyzed at the 6-month time point. Second, the study exclusively used cases that displayed disseminated lesions, while our study included all LD patients that presented with a 5 cm or larger EM rash. Interestingly, our study includes 24 cases displaying disseminated lesions and we did not observe a relationship between lesion features and transcriptional clustering (Table 2). We suggest that the differences in outcomes between studies may be due to variation in clinical case definition, sample size and potentially differences in *Borrelia burgdorferi sensu stricto* strains that initiate disease in differing geographic regions.

**Table 2:** Baseline demographic and clinical characteristics of 72 participants with early Lyme disease by v1 cluster group^a^.

	**Whole Sample**	**Cluster 0**	**Cluster 1**	**Cluster 2**	**Overall *p*-value**	**0 vs. 1 *p*-value**	**0 vs. 2 *p*-value**	**1 vs. 2 *p*-value**
	***n* = 72^b^**	***n* = 40**	***n* = 19**	***n* = 13**				
Age (years)	49.5 [33.0–60.0] (20.0–77.0)	49.0 [31.5–57.5] (21.0–73.0)	53.0 [31.0–64.0] (20.0–71.1)	50.0 [42.0–61.0] (23.0–77.0)	0.649	0.434	0.504	0.939
Female gender	31 (43.1%)	16 (40.0%)	8 (42.1%)	7 (53.9%)	0.678	0.878	0.382	0.513
Erythema migrans size (cm^2^)	82.0 [50.0–157.0] (16.0–900.0)	99.5 [52.0–169.0] (16.0–375.0)	84.0 [48.0–144.0] (32.0–900.0)	60.0 [50.0–126.0] (24.0–182.0)	0.505	0.646	0.248	0.569
Disseminated erythema migrans	22 (30.6%)	12 (30.0%)	5 (26.3%)	5 (38.5%)	0.760	0.770	0.734	0.699
Duration of illness (days)	6.0 [4.0–10.0] (1.0–60.0)	6.0 [4.0–9.5] (1.0–60.0)	5.0 [4.0–16.0] (3.0–42.0)	6.0 [3.0–6.0] (3.0–13.0)	0.535	0.727	0.322	0.359
Antibiotic treatment initiated at V1^c^	27 (37.5%)	21 (52.5%)	4 (21.1%)	2 (15.4%)	0.013	0.022	0.019	1.000
Number of new onset, Lyme-related symptoms	6.0 [3.0–10.0] (0.0–26.0)	8.5 [4.0–14.0] (0.0–26.0)	6.0 [1.0–8.0] 0.0–13.0)	5.0 [3.0–6.0] (1.0–10.0)	0.025	0.025	0.052	0.924
Absolute lymphocytes <1.1 × 10^3^/μL	14 (19.4%)	8 (20.0%)	2 (10.5%)	4 (30.8%)	0.331	0.476	0.459	0.194
Liver function abnormality^d^	22 (30.6%)	15 (37.5%)	6 (31.6%)	1 (7.7%)	0.127	0.657	0.079	0.195
Two-tier antibody positive (acute)	18 (25.0%)	13 (32.5%)	3 (15.8%)	2 (15.4%)	0.322	0.177	0.305	1.000
Two-tier antibody positive	31 (43.1%)	22 (55.0%)	5 (26.3%)	4 (30.8%)	0.071	0.039	0.129	1.000
**PCR/ESI-MS^e^ results:**								
Skin (+)/blood (+)	19/69 (27.5%)	12/39 (30.8%)	5 (26.3%)	2 (18.2%)	0.925	0.936	0.690	0.892
Skin (+)/blood (–)	29/69 (42.0%)	15/39 (38.5%)	8 (42.1%)	6 (54.6%)				
Skin (–)/blood (–)	21/69 (30.4%)	12/39 (30.8%)	6 (31.6%)	3 (27.3%)				

a *Data from categorical variables are presented as count (%). Data from normally distributed variables are presented as mean ± standard deviation (range) and from continuous variables without normal distribution as median (25th percentile, 75th percentile) (range). Comparisons by cluster group were performed using Kruskall-Wallis tests for overall p-value and Wilcoxon rank sum test for individual pairwise comparisons for continuous variables, and chi-square test for categorical variables*.

b *N = 73 participants were included in this study. However, one did not have a V1 blood draw and therefore V1 cluster status could not be determined and they were dropped from this analysis*.

c *Participants were eligible if they had initiated appropriate antibiotic treatment for Lyme disease <72 h from their baseline study visit*.

d *Elevated liver function test defined as any one of the following: aspartate aminotransferase above 35 U/L, alanine transaminase above 40 U/L, or alkaline phosphatase above 130 U/L for males and above 115 U/L for females*.

e *PCR and electrospray ionization mass spectrometry (39). Three participants were missing PCR/ESI-MS results*.

In our study, dimensionality reduction followed by clustering analysis of the longitudinal RNA-seq data identified three distinct clusters. In one cluster, containing mostly cases, the patients can be observed mixing with another cluster, containing mostly controls, over multiple visits. However, patients within another cluster containing mostly cases, stay in the same region of expression space even at later visits after treatment. This pattern of remaining within a cluster over time suggests that there is a long-term alteration of genes targeted for transcription in PBMCs following acute LD diagnosis. However, it is still unclear what is driving this persistent transcriptional activity. Possibilities include the continued presence of spirochetes or foreign bacterial antigen as well as epigenetic changes, all of which could drive long term transcriptional alterations. It is interesting to point out that for a subset of 21 cases, Borrelia DNA was not detected in the skin or blood using extremely sensitive PCE/ESI-MS based approach. Initially, we categorized these cases as potential cases of STARI, or sampling or diagnostic errors. However, in this current study, most of these patients have transcriptional profiles that cluster them with other cases, suggesting that these may represent true LD cases for which the applied detection methodologies failed. This may also suggest that profiling host gene expression may be a useful and potentially a sensitive approach for the diagnosis of acute LD.

The three clusters defined by the DEGs also have distinct clinical and immunological features. Cluster 0, the largest cluster, was found to have a higher number of symptoms of acute disease, higher rates of two-tier antibody positivity, as well as a non-statistically significant trend toward higher rates of abnormal liver function tests. All together, these imply that this cluster has more severe acute disease and a robust immune response. This is consistent with the enrichment analysis, which associates cluster 0 with immune mediated common and rare diseases. Also revealed by the enrichment analysis, was an association with CD8+ T cells, a subset usually linked to the control of intracellular pathogens via cytotoxic effector function. Interestingly, CD8+ T cells have been implicated in LD (40, 41). Cluster 0 also uniquely displays elevated levels of IL-31, member of the gp130/IL-6 family of cytokines (42). This cytokine is thought to be produced by CD4+ Th2 cells but Th1 cells can be induced to express IL-31 (43). IL-31 is also known to promote skin inflammatory disorders in mouse models and in the human setting (42, 44). Interestingly, in a retrospective study, the development of systemic autoimmune joint diseases, including psoriatic arthritis was associated with prior LD (45). Also, in our study, differential gene expression analysis comparing the controls to the cases identify immune response genes that may be specific for LD. Enriched terms include LD common symptoms such as erythema and arthritis. The overlapping genes that lead to the significant overlap between the differentially expressed genes and these terms may directly suggest novel molecular mechanisms of disease. Cluster 1 is likewise distinct as these cases have milder disease. In addition, enrichment analysis associates this cluster with distinct cells, pathway and processes including neutrophils, IL-4 signaling, IgA/IgE production as well a novel and rare diseases which are thought not to involve immune mediated processes. Neutrophils are thought not to be a major effector cell in LD, although Osp A can activate human neutrophils (46). The notion that infection with *B. burgdorferi* may trigger such non-immune pathways is an intriguing idea that may help explain the range of disease outcomes linked to LD. Previous work found that the human immune response in LD is largely driven by monocytes and Th1 driven mechanisms (47, 48). However, these studies largely focused on T cells recovered from inflamed joints in Lyme arthritis, a late feature of untreated LD. Our studies indicate that a wide range of immune effector cells may be engaged in LD including NK cells, CD8+ T cells and neutrophils, which may help define disease subgroups and inform treatment strategies. Clearly, future studies involving detailed immune profiling of multiple LD cohorts are required.

Analysis of our gene expression data by CIBERSORT revealed an enriched signal for Treg cells in Lyme cases. T regulatory cells have well established roles in the regulation of self-immunity as well as influencing the outcomes of infection (49). In human Lyme disease, low levels of Tregs in the synovial fluid were associated with a longer duration of illness in antibiotic-refractory Lyme arthritis, a late manifestation of human borrelliosis (50, 51). This is also consistent with the finding that *in vivo* depletion of Tregs accelerated inflammatory arthritis in a mouse model (52), suggesting that Tregs in the context of Lyme arthritis exhibit anti-inflammatory properties. The significance of the elevated Treg signal in our study of acute Lyme disease is not clear. Several studies have shown that in human tuberculosis increased Tregs levels are associated with active disease, suggesting that in some cases Treg responses may impair pathogen clearance and disease resolution (53, 54). Whether this is the case in some stages of human Lyme disease, or if these cells contribute to the observed dysregulated or maladaptive immune response in Lyme disease (55, 56), will require additional studies on the identification and characterization of T regs in various stages and outcomes of Lyme disease. This can be potentially achieved via single cell RNA-sequencing.

## Data Availability Statement

The datasets presented in this study can be found in online repositories. The names of the repository/repositories and accession number(s) can be found below: https://github.com/LymeMIND/LM2-study-supporting-materials/tree/main/data.

## Ethics Statement

The studies involving human participants were reviewed and approved by JHU & ISMMS IRB. The patients/participants provided their written informed consent to participate in this study.

## Author Contributions

AR, JF, MWE, MJM, WR, NR, MS, and JA collected the data. DC, AR, AB, MW, MD, and AM performed the analyses. DC, AR, AB, SJ, JB, MS, JA, and AM wrote the paper. All authors contributed to the article and approved the submitted version.

## Conflict of Interest

MWE and MJM were employees of Ibis Biosciences, an Abbott Company, which developed the PCR/ESI-MS assays and instrumentation used in these studies; assays described are for research use only. The remaining authors declare that the research was conducted in the absence of any commercial or financial relationships that could be construed as a potential conflict of interest.
