# Supplementary material for: Predicting Lyme Disease From Patients' Peripheral Blood Mononuclear Cells Profiled With RNA-Sequencing
Source: Front Immunol. 2021 Mar 8;12:636289. doi: 10.3389/fimmu.2021.636289 (PMC7982722; doi:10.3389/fimmu.2021.636289)
Supplement: Supplementary file 1 [file Data_Sheet_1.pdf]

Supplementary Material

1 Supplementary Figures

A

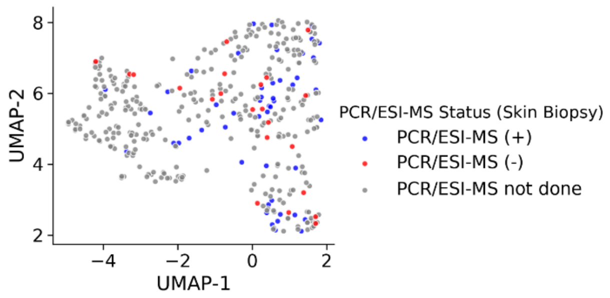

B

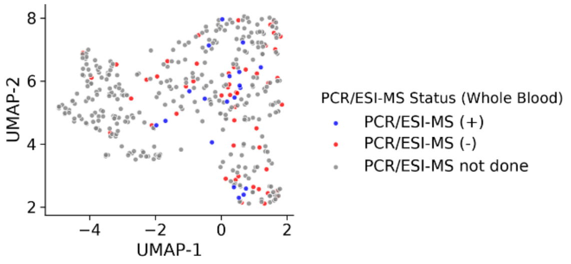

Supplementary Figure 1. UMAP projection of all samples collected for the study based on RNA-seq expression where each sample, representing a patient at a specific visit, is colored by positive (blue), negative (red) detection of antigens via PCR/ESI-MS in the (A) skin or (B) blood. Samples marked in gray did not have this test done. This test was applied to 68/72of the patients during their first visit.

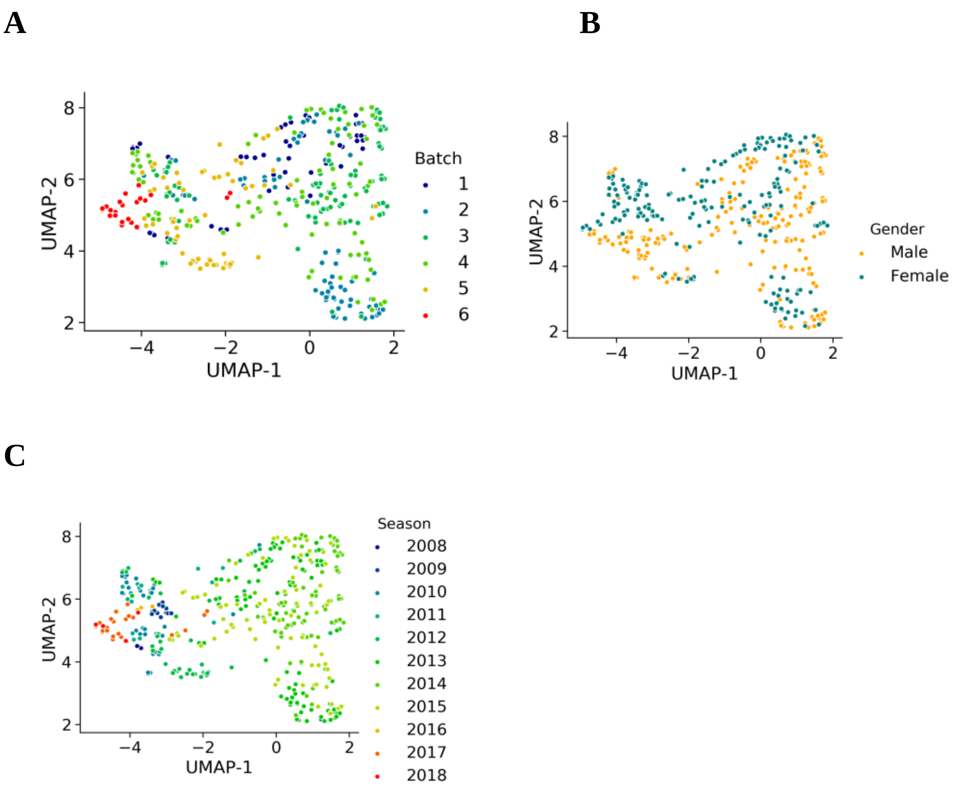

Supplementary Figure 2. UMAP projection of all samples collected for the study based on RNA-seq expression where each sample, representing a patient at a specific visit, is colored by (A) batch; (B) gender; and (C) season.

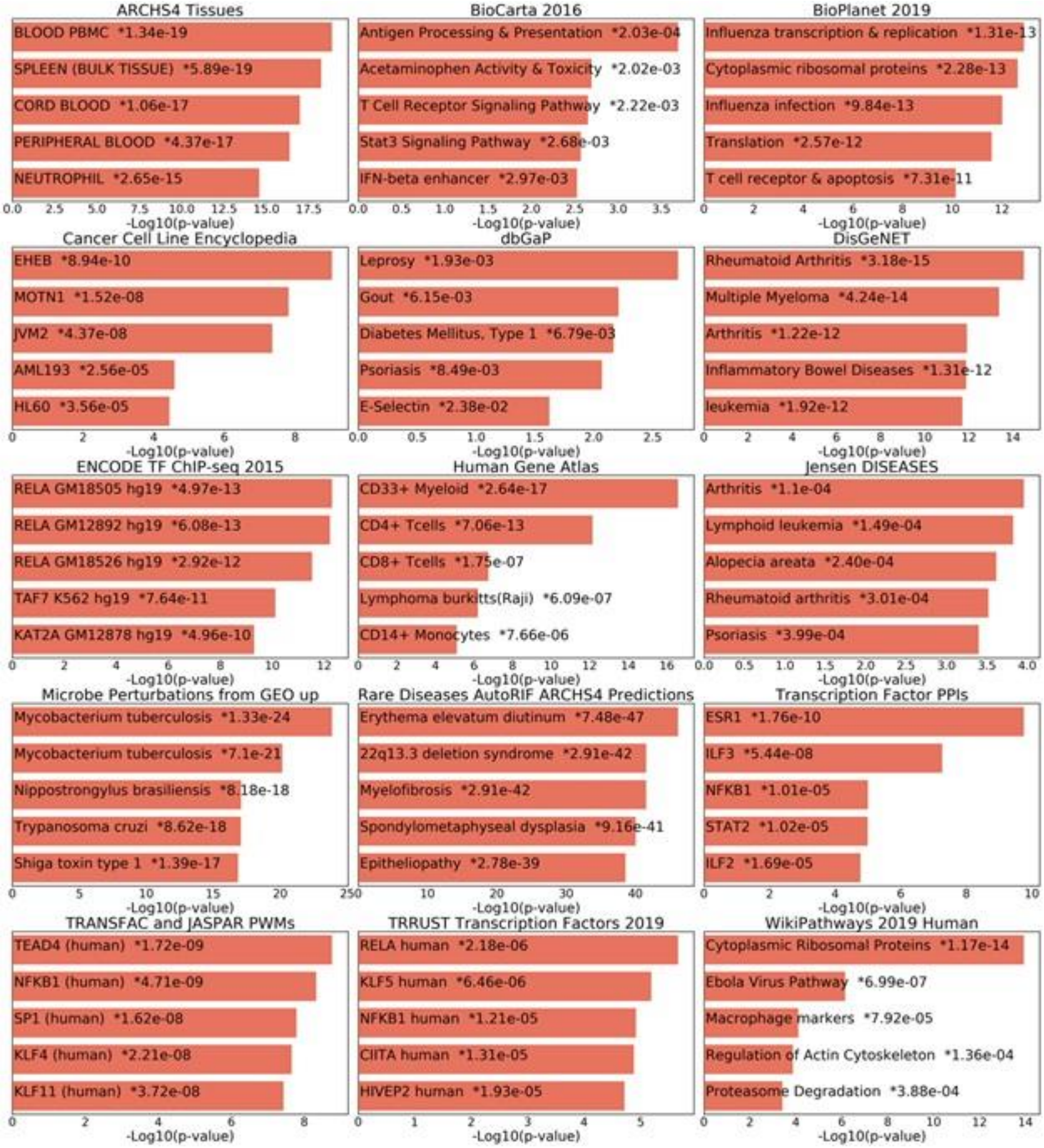

Supplementary Figure 3. Top 5 enriched terms in multiple gene set libraries for the top 200 up-regulated genes in LD cases based on Enrichr analysis.

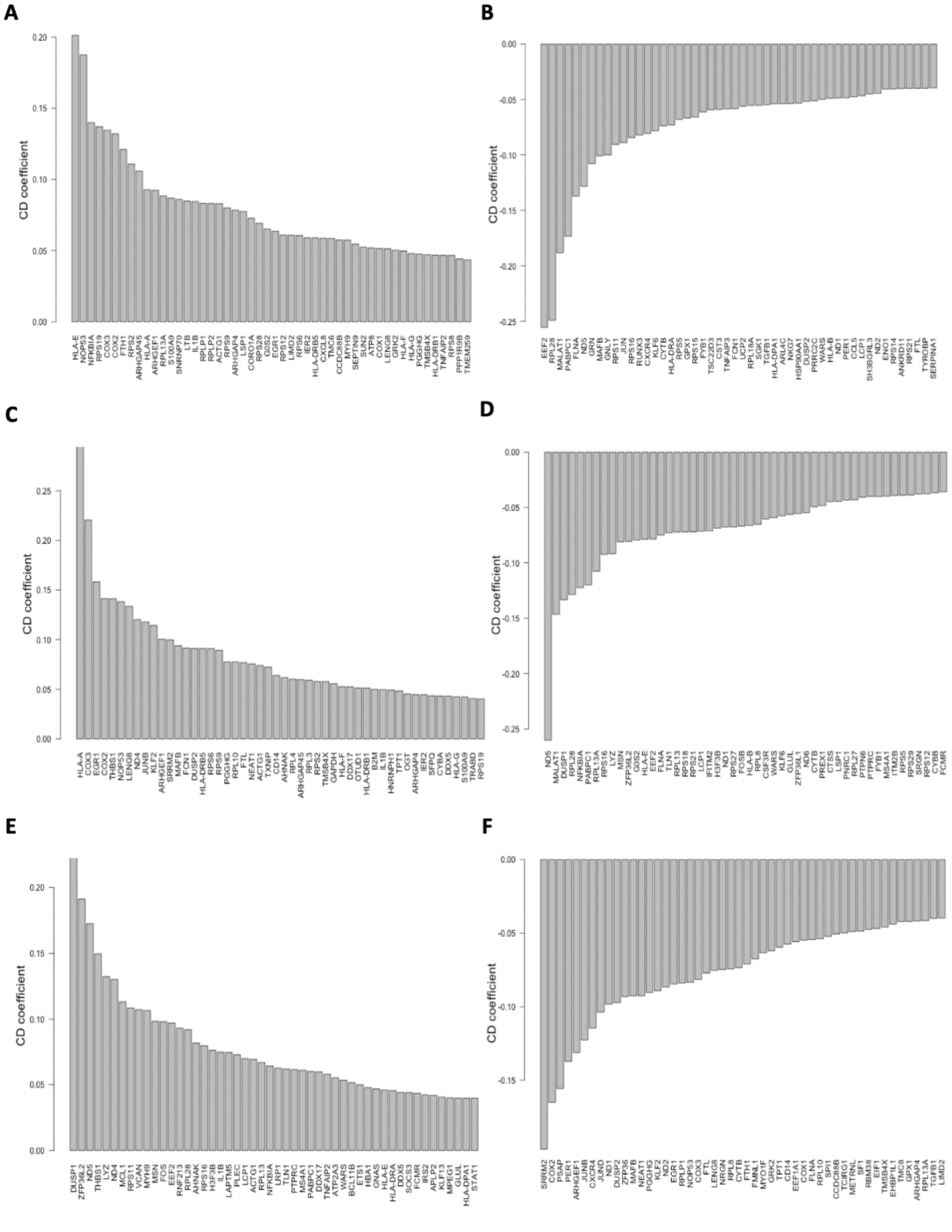

Supplementary Figure 4. Top ranked up-regulated and down-regulated differentially expressed genes between all pairwise comparisons of clusters based on the Characteristic Direction method. (A) Cluster 1 Up-regulated Genes (vs. Cluster0) / Cluster 0 Down-regulated Genes (vs. Cluster 1). (B) Cluster 1 Down-regulated Genes (vs. Cluster 0) / Cluster 0 Up-regulated Genes (vs. Cluster 1). (C) Cluster 1 Up-regulated Genes (vs. Cluster 2) / Cluster 2 Down-regulated Genes (vs. Cluster 1). (D) Cluster 1 Down-regulated Genes (vs. Cluster 2) / Cluster 2 Up-regulated Genes (vs. Cluster 1). (E) Cluster 2 Up-regulated Genes (vs. Cluster 0) / Cluster 0 Down-regulated Genes (vs. Cluster 2). (F) Cluster 2 Down-regulated Genes (vs. Cluster 0) / Cluster 0 Up-regulated Genes (vs. Cluster 2).

A

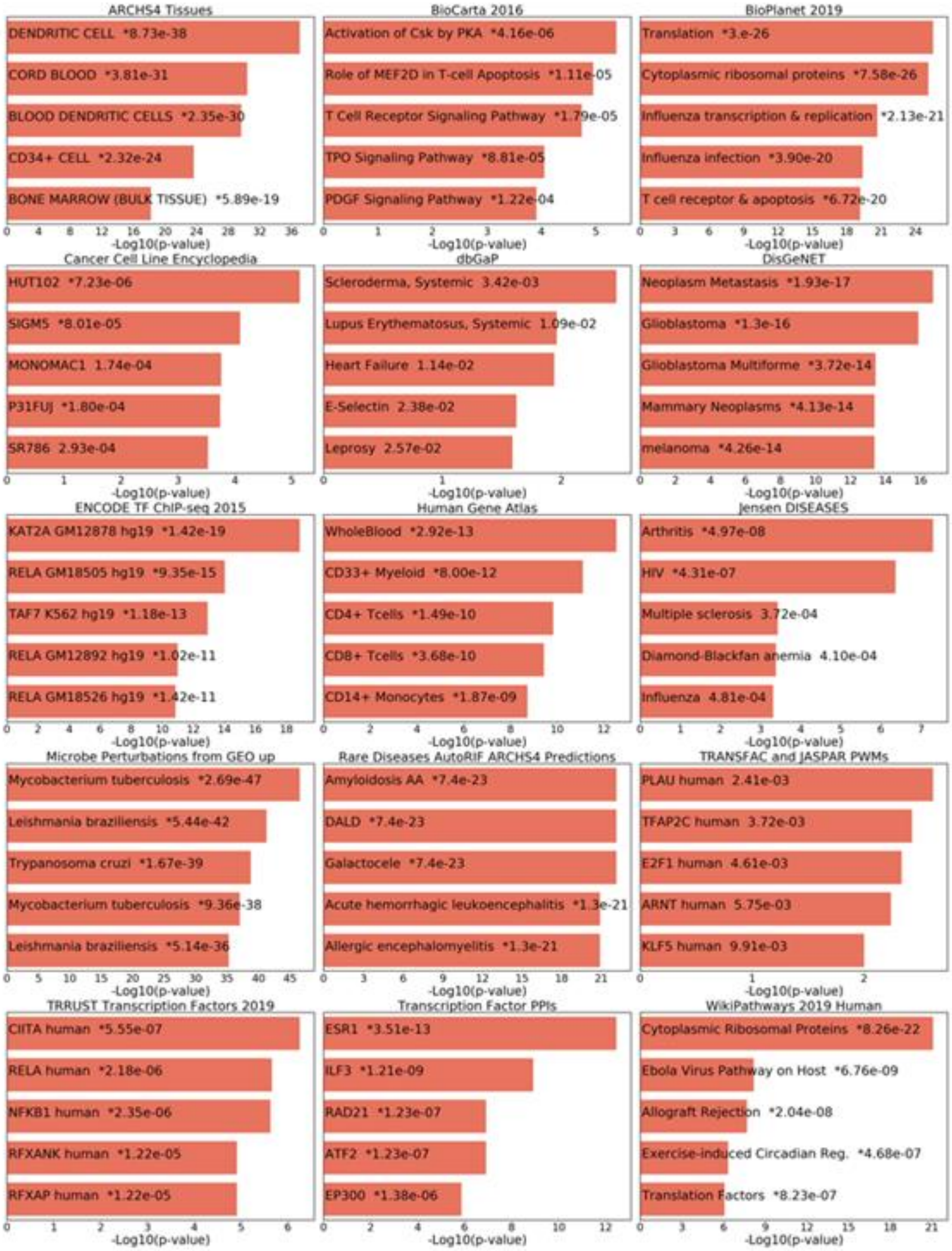

ARCHS4 Tissues
DENDRITIC CELL *8.73e-38
CORD BLOOD *3.81e-31
BLOOD DENDRITIC CELLS *2.35e-30
CD34+ CELL *2.32e-24
BONE MARROW (BULK TISSUE) *5.89e-19
-Log10(p-value)
BioCarta 2016
Activation of Csk by PKA *4.16e-06
Role of MEF2D in T-cell Apoptosis *1.11e-05
T Cell Receptor Signaling Pathway *1.79e-05
TPO Signaling Pathway *8.81e-05
PDGF Signaling Pathway *1.22e-04
-Log10(p-value)
BioPlanet 2019
Translation *3.e-26
Cytoplasmic ribosomal proteins *7.58e-26
Influenza transcription & replication *2.13e-21
Influenza infection *3.90e-20
T cell receptor & apoptosis *6.72e-20
-Log10(p-value)
Cancer Cell Line Encyclopedia
HUT102 *7.23e-06
SIGM5 *8.01e-05
MONOMAC1 1.74e-04
P31FUJ *1.80e-04
SR786 2.93e-04
-Log10(p-value)
dbGaP
Scleroderma, Systemic 3.42e-03
Lupus Erythematosus, Systemic 1.09e-02
Heart Failure 1.14e-02
E-Selectin 2.38e-02
Leprosy 2.57e-02
-Log10(p-value)
DisGeNET
Neoplasm Metastasis *1.93e-17
Glioblastoma *1.3e-16
Glioblastoma Multiforme *3.72e-14
Mammary Neoplasms *4.13e-14
melanoma *4.26e-14
-Log10(p-value)
ENCODE TF ChIP-seq 2015
KAT2A GM12878 hg19 *1.42e-19
RELA GM18505 hg19 *9.35e-15
TAF7 K562 hg19 *1.18e-13
RELA GM12892 hg19 *1.02e-11
RELA GM18526 hg19 *1.42e-11
-Log10(p-value)
Human Gene Atlas
WholeBlood *2.92e-13
CD33+ Myeloid *8.00e-12
CD4+ Tcells *1.49e-10
CD8+ Tcells *3.68e-10
CD14+ Monocytes *1.87e-09
-Log10(p-value)
Jensen DISEASES
Arthritis *4.97e-08
HIV *4.31e-07
Multiple sclerosis 3.72e-04
Diamond-Blackfan anemia 4.10e-04
Influenza 4.81e-04
-Log10(p-value)
Microbe Perturbations from GEO up
Mycobacterium tuberculosis *2.69e-47
Leishmania braziliensis *5.44e-42
Trypanosoma cruzi *1.67e-39
Mycobacterium tuberculosis *9.36e-38
Leishmania braziliensis *5.14e-36
-Log10(p-value)
Rare Diseases AutoRIF ARCHS4 Predictions
Amyloidosis AA *7.4e-23
DALD *7.4e-23
Galactocele *7.4e-23
Acute hemorrhagic leukoencephalitis *1.3e-21
Allergic encephalomyelitis *1.3e-21
-Log10(p-value)
TRANSFAC and JASPAR PWMs
PLAU human 2.41e-03
TFAP2C human 3.72e-03
E2F1 human 4.61e-03
ARNT human 5.75e-03
KLF5 human 9.91e-03
-Log10(p-value)
TRRUST Transcription Factors 2019
CIITA human *5.55e-07
RELA human *2.18e-06
NFKB1 human *2.35e-06
RFXANK human *1.22e-05
RFXAP human *1.22e-05
-Log10(p-value)
Transcription Factor PPIs
ESR1 *3.51e-13
ILF3 *1.21e-09
RAD21 *1.23e-07
ATF2 *1.23e-07
EP300 *1.38e-06
-Log10(p-value)
WikiPathways 2019 Human
Cytoplasmic Ribosomal Proteins *8.26e-22
Ebola Virus Pathway on Host *6.76e-09
Allograft Rejection *2.04e-08
Exercise-induced Circadian Reg. *4.68e-07
Translation Factors *8.23e-07
-Log10(p-value)

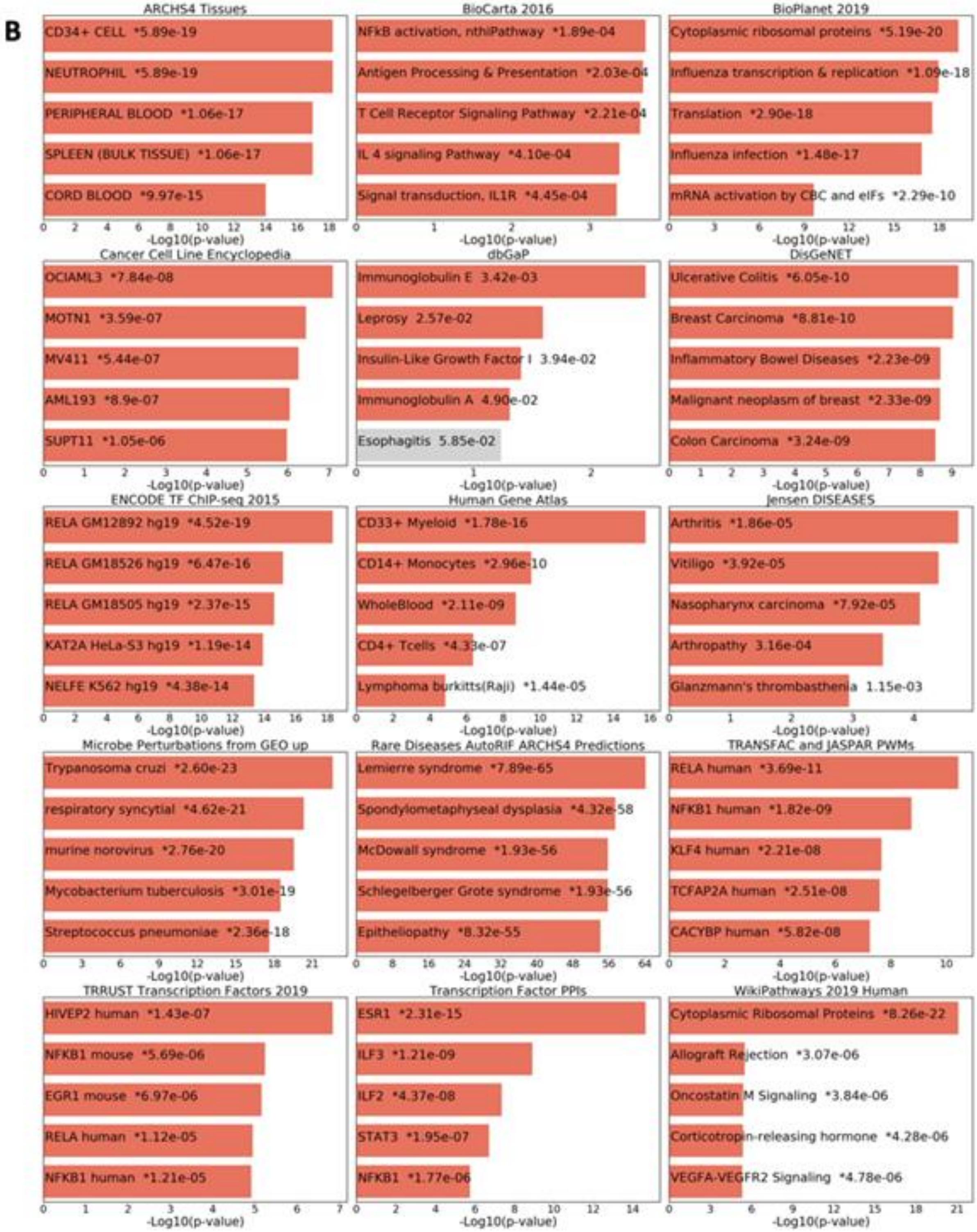

Supplementary Figure 5. Top 5 enriched terms in multiple gene set libraries based on Enrichr analysis for the top 200 up-regulated genes in (A) cluster 0 when compared to cluster 1 and (B) cluster 1 when compared to cluster 0.
